# The work engagement and organizational silence among nurses: the mediating role of coworker support

**DOI:** 10.3389/fpubh.2025.1660100

**Published:** 2025-11-21

**Authors:** Ying Shen, Xiaodan Chen, Heran Zhang, Xiangyan Lv

**Affiliations:** 1 Department of Critical Care Medicine, The Second Affiliated Hospital of Zhejiang Chinese Medical University, Hangzhou, China; 2 Nursing Department, The Second Affiliated Hospital of Zhejiang Chinese Medical University, Hangzhou, China

**Keywords:** organizational silence, work engagement, coworker support, mediating role, nurses

## Abstract

**Introduction:**

Organizational silence is prevalent in healthcare and negatively affects nurses and organizational development. This study determined whether coworker support mediates the relationship between organizational silence and work engagement among nurses.

**Methods:**

A quantitative cross-sectional survey was conducted using convenience sampling. The Utrecht Work Engagement Scale-17, Peer Supporting Scale, and Employee Silence Behavior Survey Questionnaire were used to measure the key variables. Descriptive statistics, Pearson correlation analysis, and a structural equation modeling with bootstrap method were performed.

**Results:**

A total of 597 registered nurses from 21 general hospitals in China participated. Nurses’ work engagement (72.09 ± 20.33), coworker support (108.60 ± 20.66), and organizational silence (32.23 ± 11.06) were at moderate levels. Work engagement was positively correlated with coworker support, while both work engagement and coworker support were negatively correlated with organizational silence (all *p* < 0.01). Mediation analysis indicated that the direct effect value of work engagement on organizational silence was −0.155 (95% CI: −0.217 ~ −0.094, *p* < 0.001). The indirect effect value of work engagement on organizational silence through coworker support was −0.197 (95% CI: −0.236 ~ −0.160, *p* < 0.001), accounting for 56.13% of the total effect (−0.351; 95%CI: −0.410 ~ −0.292, *p* < 0.001).

**Conclusion:**

Work engagement was negatively correlated with organizational silence, in which coworker support played a partial mediating role. It is recommended to enhance the positive impact of work engagement on organizational behavior through strengthening coworker support among nurses, thereby reducing organizational silence, fostering a better work environment, and ultimately enhancing the quality of nursing care.

## 1 Introduction

Organizational silence is defined as the intentional withholding of ideas, concerns, or information related to organizational improvement by employees (1). This phenomenon is prevalent in healthcare institutions (2). However, due to its nature as an absence of verbal expression and observable behavior, it is often inconspicuous and difficult for managers to detect, making it easily overlooked by the organization. Prolonged organizational silence can exert negative impacts on both individuals and the organization. At the individual level, when nurses’ voices are ignored or their concerns remain unaddressed, they may experience increased burnout (3), decreased job satisfaction (4), lower job performance (5), and even elevated turnover intention (6). At the organizational level, nurses’ silence can hinder managers’ ability to make timely and accurate decisions, reduce the efficiency and quality of organizational decision-making, and impede team development and innovation (7). Moreover, Jones and Durbridge (8) and Okuyama et al. (9) have reported that organizational silence among healthcare staff may jeopardize patient safety. Therefore, understanding the mechanisms that reduce nurses’ organizational silence is crucial for improving nurses’ job satisfaction, decreasing turnover intention, and ensuring the quality of nursing care. Additionally, it plays an important role in promoting hospital innovation and sustainable development within healthcare institutions.

Existing research indicates that the emergence of organizational silence is influenced by multiple factors at the individual [e.g., character, seniority, self-efficacy, work engagement (10, 11)], organizational [e.g., leadership style, organizational climate, coworker support (12–14)], and societal levels [e.g., hierarchy and national culture (15)]. Among these, work engagement and coworker support represent two significant influencing factors. Coworker support refers to the emotional concern, informational assistance, and tangible aid provided by colleagues, constituting a key dimension of social support (16). Meanwhile, work engagement—a positive psychological state characterized by vigor, dedication, and absorption (17)—has been found to be a critical personal resource that enhances performance and career satisfaction (18–20).

Previous studies have demonstrated that both work engagement and coworker support are negatively correlated with organizational silence (21). Furthermore, Li et al. (22), using structural equation modeling, have elucidated the mechanism through which work engagement influences silence behavior: nurses with higher levels of work engagement were more likely to break the silence and proactively voice their opinions and ideas to the organization. It has also been observed that nurses’ work engagement may be closely associated with support from nurse supervisors (23). However, no study to date has examined the role of coworker support in the pathway between work engagement and silence behavior among nurses. Given the importance of teamwork and peer support in nursing practice (24, 25), it is essential to investigate the underlying mechanisms linking these three constructs.

The Conservation of Resources (COR) theory was proposed by American psychologist Hobfoll (26) in 1989. It predicts behavior from a resource-based perspective, explaining not only motivation but also the resource-related conditions under which behavior occurs (27). This theoretical framework provides a valuable basis for understanding individuals’ behavioral choices in resource-limited contexts. Therefore, the present study employed COR theory to explore the relationships among nurses’ work engagement, coworker support, and organizational silence.

First, COR theory categorizes resources into four dimensions: object resources (e.g., shelter and clothing), personal characteristic resources (e.g., personality traits and coping abilities), condition resources (e.g., social relationships, job status, and health), and energy resources (e.g., time, knowledge, and skills) (28). In this study, work engagement and coworker support are conceptualized as personal characteristic and conditional resources, respectively. Second, the basis of COR theory is that individuals are motivated to conserve, maintain, and acquire resources that they value (26). When any of these four resources are threatened or lost, individuals experience stress. Individuals with abundant resources are more capable of acquiring new resources and are more willing to invest them, whereas those with scarce resources tend to adopt defensive strategies to prevent further loss (26). Moreover, the theory explains behavior through the lens of imbalance between resource investment and return: when resource investment fails to yield expected returns, individuals are likely to exhibit negative behavioral responses; conversely, when investment results in satisfactory returns, individuals are motivated to engage in positive behaviors (26).

**Figure 1:**
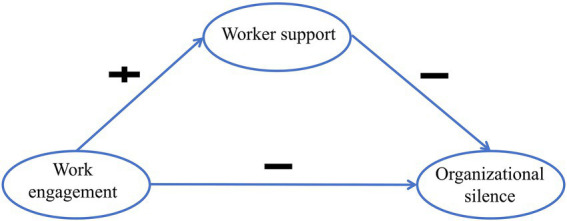
Hypothetical model of this study.

Based on COR theory, this study proposes the following hypotheses: (1) Nurses with more personal characteristic resources (higher work engagement) are less likely to adopt resource-conservation strategies (organizational silence). Thus, work engagement is negatively associated with organizational silence. (2) Nurses with more conditional resources (higher coworker support) are less likely to adopt resource-conservation strategies (organizational silence). Thus, coworker support is negatively associated with organizational silence. (3) Nurses with abundant personal characteristic resources (higher work engagement) are more likely to acquire new conditional resources (higher coworker support). Thus, work engagement is positively associated with coworker support. (4) When nurses’ investment of personal characteristic resources (work engagement) leads to conditional resource returns (coworker support), they are more likely to engage in proactive behaviors such as speaking up; conversely, inadequate resource returns or losses may increase the likelihood of organizational silence. Thus, coworker support serves as a mediating factor between work engagement and organizational silence. The theoretical framework of this study is presented in Figure 1.

The aim of this study was to examine the relationships among nurses’ work engagement, coworker support, and organizational silence, and to verify the mediating effect of coworker support between work engagement and organizational silence.

## 2 Methods

### 2.1 Study design and setting

This quantitative cross-sectional study was conducted between January and March 2024 at 21 general hospitals in Hangzhou, China.

### 2.2 Participants

Registered nurses from the 21 hospitals were recruited through convenience sampling. Eligible participants were those aged 18 years or older and holding at least an associate nursing degree. Nurses who were interns, on rotation or standardized training, pursuing further education, or on sick or maternity leave were excluded from the study.

### 2.3 Sample size

The sample size was determined based on structural equation model (SEM) parameter considerations. In the hypothesized model, we estimated 11 variance parameters, 4 direct and indirect path coefficients among latent variables, and 8 factor loadings, yielding a total of 23 free parameters. Following the rule of at least 10 observations per free parameter for SEM (29, 30), a minimum of 230 participants was required. Considering 10% of invalid response rate, the final required sample size was set at 256.

### 2.4 Measurements

1. Demographic information: demographic characteristics encompassed sex, age, educational background, department, professional title, position, and marital status.
2. The Chinese version of Utrecht Work Engagement Scale (UWES-17): the Chinese version of UWES-17, adapted by Zhang and Gan (31), was used to assess nurses’ work engagement in this study. The Chinese version includes three subscales: vigor (6 items), dedication (5 items), and absorption (6 items), using a 7-point Likert scale, with higher scores indicating higher levels of work engagement. The overall Cronbach’s *α* coefficient of the scale was approximately 0.9 (31). In this study, the Cronbach’s α of the scale was 0.954.
3. Peer Supporting Scale (PSS): this scale was developed by Ye et al. (32), based on the support scale created by Greene et al. (54), and adapted for the Chinese nursing population. The scale consists of two parts. Scale A is the Nurse Supervisor Support Scale, comprising 9 items, which reflects nurses’ evaluation of the support provided by their nurse supervisors. Scale B, which contains 21 items, measures nurses’ perceptions of peer support among colleagues and is subdivided into seven dimensions: subjective support, collaboration, empathy, awareness enhancement, goal setting, action planning, and process management evaluation. Both Scale A and B use a 5-point Likert scale, where higher scores indicate higher perceived support from colleagues. The Cronbach’s *α* coefficients for Scale A and Scale B are 0.922 and 0.959, respectively (32). In this study, the Cronbach’s *α* of the scale was 0.991.
4. Employee Silence Behavior Survey Questionnaire: this questionnaire, developed by Zheng et al. (33) in 2008 for the Chinese context, includes three dimensions: acquiescent silence, defensive silence, and disregardful silence (4 items per dimension). A 5-point Likert scale is used, with higher scores indicating a greater level of organizational silence among employees. The overall Cronbach’s *α* coefficient of the questionnaire was 0.89, and structural validity analysis showed a Kaiser-Meyer-Olkin (KMO) measure of 0.895 and a CFI of 0.94. In this study, the Cronbach’s *α* of the scale was 0.949.

### 2.5 Data collection and quality control

Data were collected between January and March 2024. The research team first contacted the nursing departments of 21 general hospitals in Hangzhou to explain the study objectives and ethical principles. After obtaining administrative approval, a designated nurse manager in each hospital assisted in distributing the online questionnaire link (created via the “Wenjuanxing” platform) to nurses through departmental WeChat groups. The survey included information about the study, an informed consent statement, and instructions for completion. Participation was voluntary and anonymous.

To ensure data quality, the questionnaire required completion of all items and limited each IP address to one submission. All responses were completed independently by participants. Two researchers reviewed and cleaned the data, excluding invalid questionnaires (e.g., those with repetitive answers or unrealistically short completion times).

### 2.6 Statistical analysis

Data were analyzed using SPSS 23.0 and AMOS 26.0. Categorical variables were presented as frequencies and percentages, while continuous variables with normal distribution were expressed as means and standard deviations. Pearson correlation analysis was used to examine the relationship between nurse work engagement, coworker support, and organizational silence.

SEM was used to test the hypothesized model (Figure 1), and mediating effects were assessed through bootstrap method with 5,000 samples and 95% bias-corrected confidence intervals (34). Confirmatory factor analysis (CFA) evaluated the reliability and validity of the measurement model. Composite reliability (CR) values above 0.70 indicated good internal consistency (35), while average variance extracted (AVE) values above 0.50 supported convergent validity (36). Discriminant validity was confirmed when the square root of each construct’s AVE exceeded its correlations with other constructs (37).

Model fit was assessed using multiple indices: *χ*^2^/df < 3, GFI, AGFI, and IFI > 0.90, and RMSEA < 0.08 indicated acceptable fit (38). Sociodemographic variables (age, education, and department) were controlled for in the mediation models. Statistical significance was set at *p* < 0.05.

## 3 Results

### 3.1 Participants

A total of 613 questionnaires were collected in this study, of which 597 were valid, resulting in an effective return rate of 97.4%. Table 1 presents the characteristics of the participants. Among the 597 nurses included in this study, 527 (88.3%) were female, and 70 (11.7%) were male, with a mean age of 31.5 ± 6.9 years (range: 20–54 years). The majority of participants held a bachelor’s degree (84.9%). Participants were primarily from general wards (48.9%) and intensive care units (30.0%), with smaller proportions from outpatient clinics (9.4%), emergency departments (6.7%), and operating rooms (5.0%). Most nurses held no official position (80.9%), and their primary title was senior nurse (45.2%). Additionally, 56.6% of the nurses were married.

**Table 1:** Baseline characteristics of the participants (*n* = 597).

Characteristics	*n*	%
Sex		
Female	527	88.3
Male	70	11.7
Educational background		
College degree	83	13.9
Bachelor’s degree	507	84.9
Master’s degree and above	7	1.2
Department		
General ward	292	48.9
Intensive care unit	179	30.0
Outpatient department	56	9.4
Emergency department	40	6.7
Operating room	30	5.0
Professional title		
Registered nurse	132	22.1
Senior nurse	270	45.2
Supervisor nurse	174	29.1
Deputy chief nurse and above	21	3.5
Position		
None	483	80.9
Responsible team leader	37	6.2
Assistant to the head nurse	6	1.0
Deputy head nurse and head nurse	29	4.9
Teaching instructor	42	7.0
Marital status		
Married	338	56.6
Unmarried	257	43.0
Others	2	0.3

### 3.2 Nurses’ scores on work engagement, coworker support, and organizational silence

The results of the self-assessment scale indicated that the total score for nurses’ organizational silence was 32.23 ± 11.06. And the mean item score was 2.69 ± 0.92. Compared to the midpoint value of 3, nurses’ organizational silence was at a moderate level. Among the dimensions, acquiescent silence had the highest mean score, while disregardful silence had the lowest. The total scores for nurses’ work engagement and coworker support were 72.09 ± 20.33 and 108.60 ± 20.66, respectively, which were also at a medium level. The specific scores are presented in Table 2.

**Table 2:** Work engagement, coworker support, and organizational silence scores among 597 nurses.

Subjects	Ranges of total score	Mean total score (x̅ ± s)	Mean score of each item (x̅ ± s)	Standardized factor loading	AVE	CR
Organizational silence, 12 items	12–60	32.23 ± 11.06	2.69 ± 0.92		0.743	0.894
Acquiescent silence, 4 items	4–20	11.58 ± 4.39	2.89 ± 1.10	0.944^***^		
Defensive silence, 4 items	4–20	11.25 ± 4.32	2.81 ± 1.08	0.898^***^		
Disregardful silence, 4 items	4–20	9.39 ± 3.69	2.35 ± 0.92	0.658^***^		
Work engagement, 17 items	0–102	72.09 ± 20.33	4.24 ± 1.20		0.936	0.978
Vigor, 6 items	0–36	25.70 ± 7.83	4.28 ± 1.30	0.888^***^		
Dedication, 5 items	0–30	22.61 ± 6.73	4.52 ± 1.35	0.936^***^		
Absorption, 6 items	0–36	23.79 ± 6.75	3.96 ± 1.12	0.966^***^		
Coworker support, 30 items	30–150	108.60 ± 20.66	3.74 ± 0.71		0.916	0.956
Scale A, 9 items	9–45	36.15 ± 7.27	4.02 ± 0.81	0.953^***^		
Scale B, 21 items	21–105	72.45 ± 13.80	3.45 ± 0.66	0.961^***^		

AVE, average variance explained; CR, construct reliability.

^***^p < 0.001.

### 3.3 Correlation analysis of nurses’ work engagement, coworker support, and organizational silence

Correlation analysis revealed that nurses’ work engagement was significantly negatively correlated with organizational silence (*r* = −0.498, *p* < 0.01). Similarly, coworker support was significantly negatively correlated with the organizational silence (*r* = −0.604, *p* < 0.01). Moreover, there was a significant positive correlation between work engagement and coworker support (*r* = 0.511, *p* < 0.01). The trend in the correlations between the dimensions was consistent with the trend between the total scores of the scales (as shown in Table 3).

**Table 3:** Correlation analysis of nurses’ work engagement, coworker support, and organizational silence.

Subjects	1	2	3	4	5	6	7	8	9	10	11
1 Organizational silence	1										
2 Acquiescent silence	0.932^**^	1									
3 Defensive silence	0.926^**^	0.850^**^	1								
4 Disregardful silence	0.808^**^	0.610^**^	0.596^**^	1							
5 Work engagement	−0.498^**^	−0.453^**^	−0.437^**^	−0.444^**^	1						
6 Vigor	−0.492^**^	−0.448^**^	−0.435^**^	−0.433^**^	0.970^**^	1					
7 Dedication	−0.502^**^	−0.453^**^	−0.442^**^	−0.451^**^	0.954^**^	0.905^**^	1				
8 Absorption	−0.430^**^	−0.393^**^	−0.373^**^	−0.386^**^	0.937^**^	0.860^**^	0.829^**^	1			
9 Coworker support	−0.604^**^	−0.613^**^	−0.550^**^	−0.439^**^	0.511^**^	0.496^**^	0.498^**^	0.468^**^	1		
10 Scale A	−0.598^**^	−0.599^**^	−0.550^**^	−0.437^**^	0.489^**^	0.472^**^	0.478^**^	0.451^**^	0.963^**^	1	
11 Scale B	−0.589^**^	−0.602^**^	−0.534^**^	−0.427^**^	0.508^**^	0.494^**^	0.495^**^	0.464^**^	0.990^**^	0.916^**^	1

^**^p < 0.01.

### 3.4 Mediation analysis of nurses’ work engagement, coworker support, and organizational silence

#### 3.4.1 Reliability and validity evidence

The composite reliability and convergent validity of the latent variables in the measurement model are shown in Table 2. Each latent variable had a CR greater than 0.70, indicating good internal consistency. All standardized factor loadings of the observational variables were greater than 0.50, and the AVE values for each latent variable exceeded 0.50, demonstrating acceptable convergent validity. As shown in Supplementary Table S1, the absolute values of the correlation coefficients between latent variables were all below 0.7, and the square root of each latent variable’s AVE was greater than its correlations with other latent variables, indicating good discriminant validity. Overall, these results suggest that the latent variables were successfully derived from their corresponding observed variables.

#### 3.4.2 Model fit

**Figure 2:**
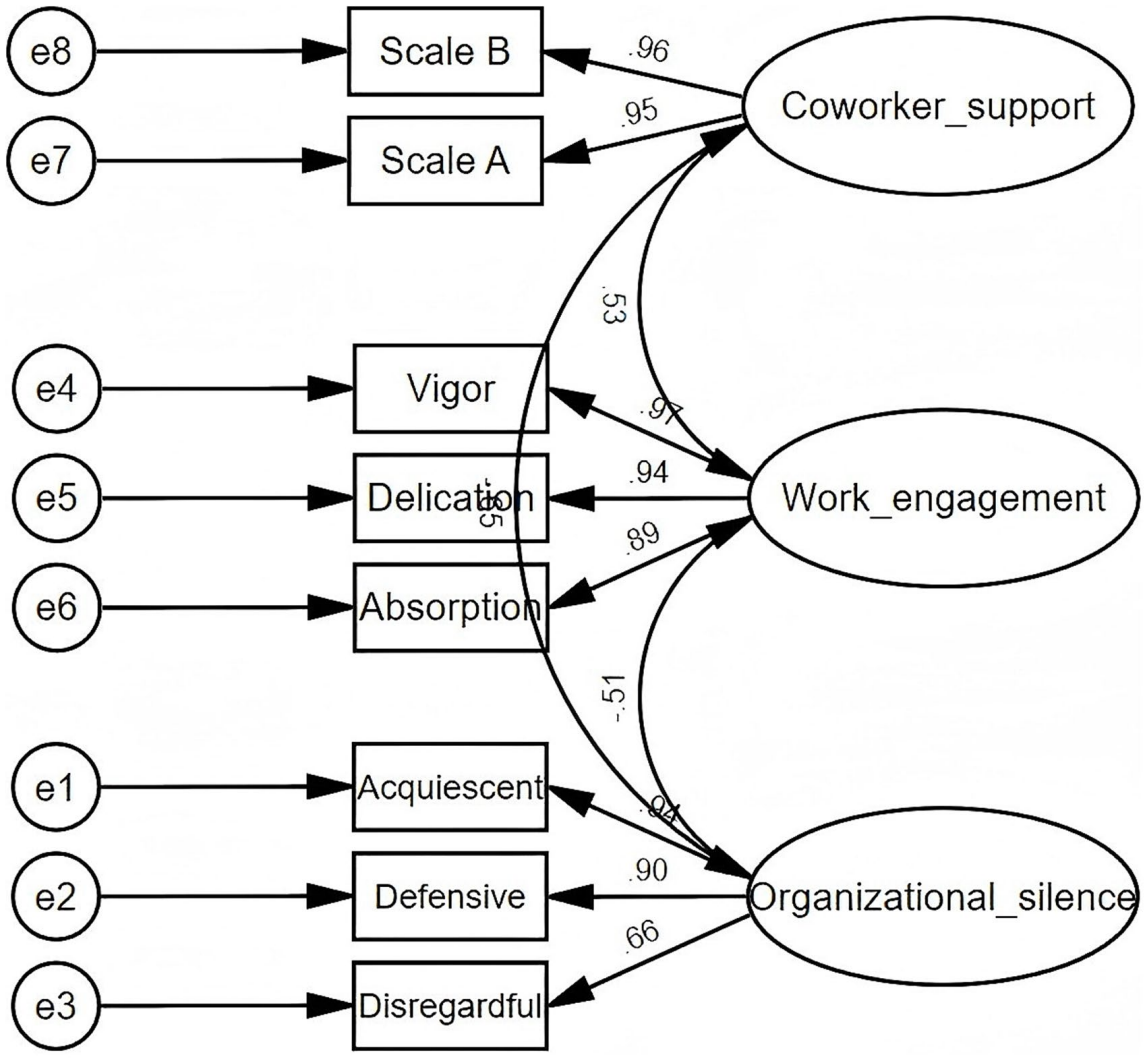
Confirmatory factor analysis of work engagement, coworker support, and organizational silence.

The measurement model developed for this study is illustrated in Figure 2. In the measurement model, the fit indices were as follows: *χ*^2^/df = 2.687 (<3), GFI = 0.982, AGFI = 0.961, IFI = 0.994 (all >0.9), and RMSEA = 0.053 (<0.08), indicating that the model demonstrated a good fit and accurately reflected the latent variables. Similarly, the structural model also showed satisfactory fit indices: *χ*^2^/df = 2.703, GFI = 0.981, AGFI = 0.961, IFI = 0.994, and RMSEA = 0.053. These results suggest that both the measurement and structural models achieved good overall fit. The established model structure is reasonable and effectively explains the path relationships among the variables.

#### 3.4.3 Hypothesis testing

**Figure 3:**
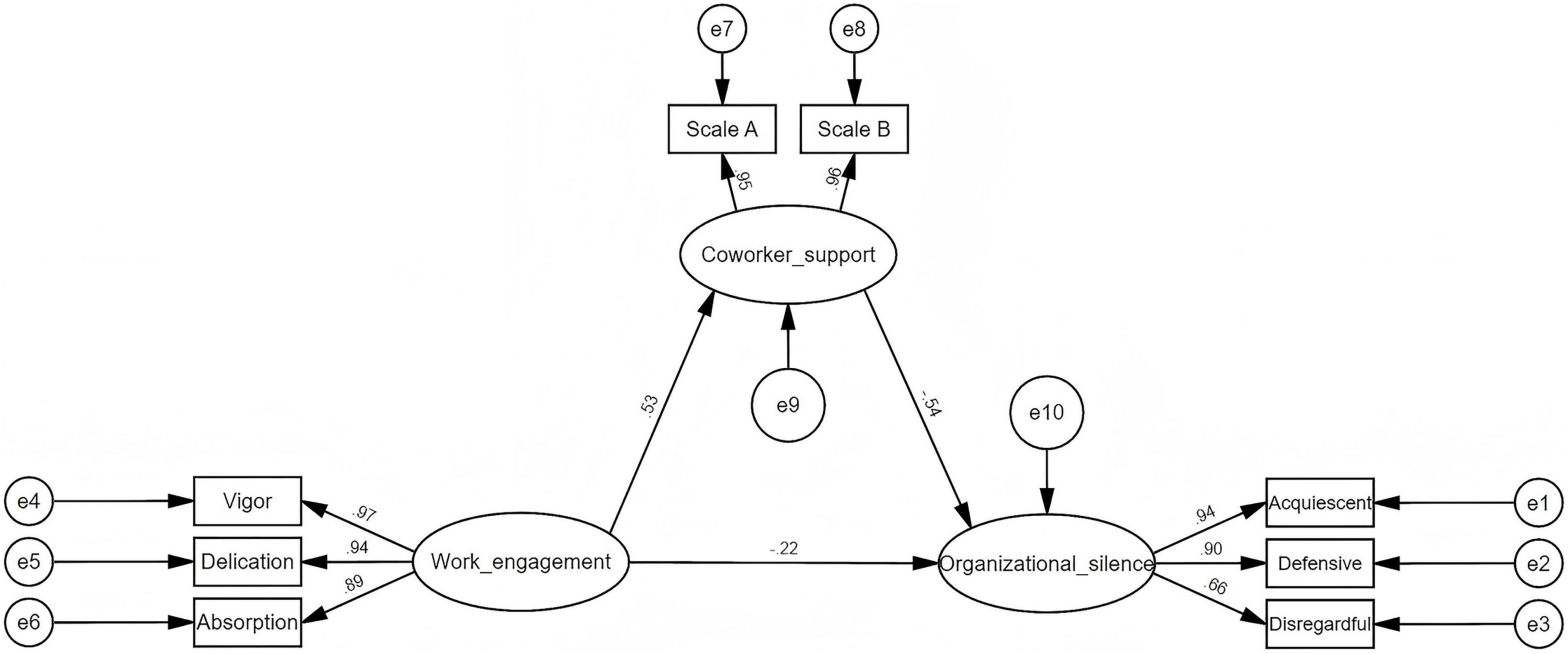
The structural equation model of work engagement, coworker support, and organizational silence.

The mediating role of coworker support was further examined by developing SEM through AMOS. SEM was constructed with work engagement as the independent variable, organizational silence as the dependent variable, and coworker support as the mediator. As shown in Figure 3, Table 4, work engagement was directly and negatively related to organizational silence (*β* = −0.22, *p* < 0.001). Work engagement was positively related to coworker support (*β* = 0.53, *p* < 0.001), while coworker support was negatively related to organizational silence (*β* = −0.54, *p* < 0.001). Additionally, coworker support partially mediated the relationship between work engagement and organizational silence. And the indirect effect (−0.197) accounted for 56.13% of the total effect (−0.351). After adjusting for the sociodemographic factors of age, education level, and department of nurses, the magnitude of the effects changed only minimally (<10%) and statistical significance remained unchanged, confirming the robustness of the results (Table 4). These findings align with the hypotheses presented in Figure 1.

**Table 4:** Mediation analysis of nurses’ work engagement, coworker support, and organizational silence.

Subjects	Estimate	Boot SE	95% CI	*p*-value
Original model				
Total effect	−0.351	0.030	(−0.410, −0.292)	<0.001
Direct effect	−0.155	0.045	(−0.217, −0.094)	<0.001
Indirect effect	−0.197	0.020	(−0.236, −0.160)	<0.001
Modified model^a^				
Total effect	−0.353	0.030	(−0.411, −0.295)	<0.001
Direct effect	−0.166	0.031	(−0.227, −0.107)	<0.001
Indirect effect	−0.187	0.020	(−0.229, −0.150)	<0.001

a Controlling for age, education level, and department of nurses.

## 4 Discussion

This study surveyed 597 nurses from 21 general hospitals in China to explore the relationship between work engagement, coworker support, and organizational silence. Consistent with our hypothesis, both work engagement and coworker support were negatively correlated with organizational silence, while work engagement was positively associated with coworker support. Moreover, coworker support was found to partially mediate the relationship between work engagement and organizational silence. These findings offer a novel theoretical perspective on organizational silence behavior among nurses and provide an evidence-based foundation for nursing management practices.

Wen et al. (39) conducted a meta-analysis involving nurses from China, South Korea, and Turkey, and reported that the average level of organizational silence among 13,394 nurses was moderate. Similarly, the nurses in this study also exhibited a moderate level of organizational silence. In contrast, studies conducted among nurses in the Philippines (40) and Egypt (41) reported slightly higher levels of organizational silence, which may be attributed to cultural or methodological differences. These findings suggested that organizational silence is a widespread phenomenon across hospitals in different countries (10). Further analysis of the three dimensions of organizational silence revealed that the mean scores ranked from highest to lowest as follows: acquiescent silence, defensive silence, and disregardful silence. This indicated that nurses’ silence is not a blind or passive behavior but rather a rational and deliberate response.

With the *β* = −0.22 and *p* < 0.001 values, the SEM results indicated a significant negative association between work engagement and organizational silence among nurses. Similar findings were reported by Lv et al. (21), who concluded that nurses with lower outpatient service, contract employment, fewer years of experience, and less work engagement were more likely to exhibit silence behavior. Conversely, other studies have interpreted this relationship in the opposite direction, suggesting that organizational silence negatively affects nurses’ work engagement. For example, from the perspective of the Job Demand-Resource model, Zhu et al. (11) argued that a culture of organizational silence restricts employees’ participation and autonomy (job demands), which may reduce their access to job resources and, consequently, lead to lower work engagement. Likewise, Yağar and Dökme Yağar (5) reported that a low-silence environment contributes to higher engagement and job performance.

Drawing on Hobfoll’s COR theory, this study provides new evidence supporting the idea that higher work engagement may help break organizational silence among nurses. We believed that highly engaged nurses tend to invest substantial cognitive and emotional resources in their work, forming a strong professional identity characterized by vigor, dedication, and absorption. This positive psychological state not only enhances professional competence but also strengthens the psychological safety required to express opinions, thereby reducing the tendency toward silence (3, 42). Supporting this view, Kaya and Eskin Bacaksiz (43) found that positive psychological capital is negatively associated with organizational silence, while numerous studies have shown a significant positive correlation between positive psychological capital and work engagement (44–46).

Another important finding of this study is that coworker support played a mediating role—accounting for 56.13% of the total effect—between work engagement and organizational silence. The positive association between work engagement and coworker support has been supported by previous research (23). Specifically, nurses with higher work engagement tend to demonstrate stronger responsibility, motivation, and collaboration, making them more likely to develop reciprocal social support networks and receive coworker support (47). However, few studies have examined the direct link between coworker support and organizational silence. To our knowledge, this may be the first study using SEM to confirm that coworker support is significantly and negatively related to nurses’ organizational silence and mediates the relationship between work engagement and silence behavior. Although prior research has not directly tested this pathway, existing evidence indicates that coworker support mediates the relationship between work engagement and proactive career behaviors (48). And higher coworker support is associated with lower burnout from work-related stressors (49). Combined with COR theory, these findings suggest that nurses can strengthen their ability to cope with stress by accumulating resources including coworker support, thereby reducing defensive behaviors such as organizational silence (26).

### 4.1 Theoretical implications

This study makes an important contribution to the theory of organizational behavior in nursing. It found that Chinese nurses exhibited a moderate level of organizational silence, providing new empirical evidence for the cross-cultural universality of this phenomenon. Most importantly, it is the first study to verify the mediating role of coworker support between work engagement and organizational silence, suggesting that highly engaged nurses can reduce silence by building reciprocal support networks. Unlike previous studies that mainly focused on the impact of toxic workplace environments on employees (50), this research reveals a pathway for reducing silence behavior among nurses and highlights the importance of horizontal coworker support in improving communication and fostering teamwork. In addition, by applying COR theory to explain nurses’ organizational silence, this study extends its applicability to the nursing context and offers a new theoretical lens for understanding the psychological mechanisms underlying silence behavior.

### 4.2 Practical implications

The findings of this study provide actionable strategies to reduce organizational silence among nurses. Firstly, healthcare institutions need to strengthen coworker support networks, including support from nurse supervisors and peers. This can be achieved by creating regular communication platforms, anonymous feedback channels, or appointing a suitable nurse as a liaison to alleviate nurses’ concerns about speaking up in a hierarchical culture (1, 51). Secondly, optimizing resource allocation and working policy is crucial. High work engagement among nurses, when not accompanied by sufficient resource support, may result in organizational silence due to excessive resource depletion. Managers can reduce overload by implementing flexible scheduling to prevent emotional resource exhaustion (52). Finally, incentive mechanisms and cultural reshaping need to be advanced simultaneously. Consistent with this study, research has shown that acquiescent silence is the most prevalent among nurses, which is closely linked to the passive compliance culture within organizations (10). Hospitals can foster an open and inclusive team atmosphere by recognizing proactive contributors or establishing a “no-punishment” error reporting system. For instance, Lee et al. (53) found in their study of Korean hospitals that nurse managers could create more supportive environments for nurses’ outspoken behaviors by inviting and valuing staff opinions and by helping physicians and senior nurses recognize the importance of all nurses’ voices.

### 4.3 Limitations

This study has several limitations. Although probability sampling is more desirable for ensuring representativeness, it would have required full administrative support across 21 general hospitals to access complete nursing rosters and considerable time and manpower for random or stratified selection, which was beyond the available resources of this unfunded project. Convenience sampling was therefore adopted in this study, as it allowed us to achieve an adequate sample size while maintaining sample heterogeneity across hospitals, departments, and professional ranks. In addition, potential bias was mitigated by controlling for confounding variables in the structural equation model. Second, all data were collected via self-reported questionnaires at a single time point, raising concerns about common method bias. While validated scales and anonymous participation were used to minimize this risk, the possibility of inflated associations among variables cannot be fully excluded. Third, the cross-sectional design prevents causal inferences among the variables. Future studies using probability-based sampling, larger and more diverse samples, multi-source data, and longitudinal designs are recommended to validate and extend these findings.

## 5 Conclusion

Work engagement was negatively associated with organizational silence, in which coworker support played a partial mediating role. It is recommended to enhance the positive impact of work engagement on organizational behavior through strengthening coworker support among nurses, thereby reducing organizational silence, fostering a better work environment, and ultimately enhancing the quality of nursing care and employee satisfaction.

## Data Availability

The original contributions presented in the study are included in the article/Supplementary material, further inquiries can be directed to the corresponding author.
