# Supplementary material for: The work engagement and organizational silence among nurses: the mediating role of coworker support
Source: Front Public Health. 2025 Nov 21;13:1660100. doi: 10.3389/fpubh.2025.1660100 (PMC12678340; doi:10.3389/fpubh.2025.1660100)
Supplement: Supplementary file 1 [file Table_1.docx]

***Supplementary Material***

1. **Discriminant Validity Testing**

We assessed the discriminant validity of the study variables based on the Fornell–Larcker criterion. As shown in Table S1, the diagonal values represent the square roots of the average variance extracted (AVE) for each latent variables, while the off-diagonal values represent the absolute values of the correlation coefficients between the latent variables. The square root of the AVE for organizational silence was 0.862, which is greater than the highest absolute correlation (0.604) with any other latent variables, indicating good discriminant validity. Similarly, work engagement and coworker support also demonstrated adequate discriminant validity.

Table S1. Discriminant validity of latent variables

| Constructs | Organizational silence | Work engagement | Worker support |
| --- | --- | --- | --- |
| Organizational silence | 0.862 |  |  |
| Work engagement | 0.498** | 0.967 |  |
| Worker support | 0.604** | 0.511** | 0.957 |
